# Interaction of Proteins Identified in Human Thyroid Cells

**DOI:** 10.3390/ijms14011164

**Published:** 2013-01-09

**Authors:** Jessica Pietsch, Stefan Riwaldt, Johann Bauer, Albert Sickmann, Gerhard Weber, Jirka Grosse, Manfred Infanger, Christoph Eilles, Daniela Grimm

**Affiliations:** 1 Plastic, Aesthetic and Hand Surgery, Otto-von-Guericke University Clinic, Leipziger Str. 44, 39120 Magdeburg, Germany; E-Mails: jessica.pietsch@med.ovgu.de (J.P.); stefan.riwaldt@st.ovgu.de (S.R.); manfred.infanger@med.ovgu.de (M.I.); 2 Max Planck Institute for Biochemistry, Am Klopferspitz 18, 82152 Martinsried, Germany; E-Mail: jbauer@biochem.mpg.de; 3 Leibniz-Institute for Analytical Sciences (ISAS), Bunsen-Kirchhoff-Str. 11, 44139 Dortmund, Germany; E-Mail: albert.sickmann@isas.de; 4 FFE Service GmbH, Frankfurter Ring 193a, 80807 Munich, Germany; E-Mail: gerhard.weber@ffeservice.com; 5 Nuclear Medicine, University Hospital Regensburg, Franz-Josef-Strauss-Allee 11, 93053 Regensburg, Germany; E-Mails: jirka.grosse@ukr.de (J.G.); sekretariat.nuklearmedizin@ukr.de (C.E.); 6 Institute of Biomedicine, Pharmacology, Aarhus University, Wilhelm Meyers Allé 4, 8000 Aarhus C, Denmark

**Keywords:** thyroid cancer cell line FTC-133, thyroid cancer cell line CGTH W-1, normal thyrocyte cells HTU-5, Random Positioning Machine (RPM), STRING network analysis

## Abstract

Influence of gravity forces on the regulation of protein expression by healthy and malignant thyroid cells was studied with the aim to identify protein interactions. Western blot analyses of a limited number of proteins suggested a time-dependent regulation of protein expression by simulated microgravity. After applying free flow isoelectric focusing and mass spectrometry to search for differently expressed proteins by thyroid cells exposed to simulated microgravity for three days, a considerable number of candidates for gravi-sensitive proteins were detected. In order to show how proteins sensitive to microgravity could directly influence other proteins, we investigated all polypeptide chains identified with Mascot scores above 100, looking for groups of interacting proteins. Hence, UniProtKB entry numbers of all detected proteins were entered into the Search Tool for the Retrieval of Interacting Genes/Proteins (STRING) and processed. The program indicated that we had detected various groups of interacting proteins in each of the three cell lines studied. The major groups of interacting proteins play a role in pathways of carbohydrate and protein metabolism, regulation of cell growth and cell membrane structuring. Analyzing these groups, networks of interaction could be established which show how a punctual influence of simulated microgravity may propagate *via* various members of interaction chains.

## 1. Introduction

The occurrence of thyroid cancer is rising faster than in other malignancies, and by the end of 2011, this tumor is expected to be the fifth most common cancer among women in the United States [1]. The most significant increases have been found in aggressive variants of the basic histotypes. Furthermore, the rate of poorly differentiated follicular thyroid cancer is currently increasing as well. Poorly differentiated or anaplastic types of thyroid cancer have an unfavorable prognosis [2] and the main problem in treating this type of tumor is its ability to develop resistance to all kinds of therapy [3]. Thus, new strategies have to be developed in order to detect novel targets for tumor therapy.

For about twelve years, we have investigated the behavior of normal and malignant thyroid cells as well as other cell types incubated on a Random Positioning Machine (RPM), a machine that simulates microgravity [4–7]. The rationale for our research is based on the assumption that understanding the behavior of cells in microgravity could help to counteract the severe impact of microgravity on humans visiting space [8,9]. Changes in protein concentrations and gene expression have been observed; we have previously reported that the expression of proteins involved in apoptosis, cell adhesion, and the extracellular matrix (ECM) is enhanced after various periods of incubation on the RPM [4]. These changes in protein contents appear to be variable, as for example, the gene expression of caspase-3 in endothelial cells cultured on an RPM was high after one hour, decreased after four days and increased again after 10 days [10]. Surprisingly, our efforts also revealed that three-dimensional multicellular spheroids developed when gravity was annulled [4]. These structures represent a simple model of a tumor, more closely resembling the *in vivo* situation than monolayer cells, but they are less complex than natural tumors [11]. Furthermore, it could be shown that the shape of three-dimensional aggregates formed on the RPM depends on the origin of the cells [12]. For several reasons, the multicellular spheroids appear worth further studies, as they might serve as pharmacological models prior to animal and clinical tests of new drugs [13,14]. Moreover, we expect to learn how cells transit from a two- to a three-dimensional growth and thus identify tumor makers as well as possibilities to drive cancer cells incorporated into three-dimensional cell assemblies into programmed cell death.

In order to gain more insight in the process of the three-dimensional multicellular spheroid formation in annulled gravity, the quantity of protein and mRNA is determined by various methods. Gene array technology and polymerase chain reaction are used to measure gene expression and mRNA production [15], respectively. The presence or absence of proteins is investigated by applying antibody-antigen reaction-based technologies, such as immuno-fluorescence staining, flow cytometry, and Western blotting [4–6]. Protein separation by free-flow isoelectric focusing (FF-IEF) and SDS-PAGE followed by mass spectrometry enables further identification of a large number of proteins [16,17]. Recently, we identified 235 types of proteins expressed in one or more of four different thyroid cell lines. Thirty-seven of these proteins were described for the first time in human thyroid cells [17].

A more profound analysis of proteins expressed by thyroid cells and detected by mass spectrometry revealed differences in protein expression between the cell lines [18]. In addition, we found that the incubation conditions of the cells either in a normal laboratory incubator or on the RPM prior to harvest influenced cellular protein expression [19,20]. Encouraged by these results, we wanted to learn more about the mechanisms of up- and down-regulation of proteins by changed gravity conditions. It was of special interest to learn how gravi-sensitive proteins, *i.e.*, proteins, whose expression is changed directly by removing gravity forces, could forward the received signal downstream. Thus, we tried to find interacting proteins as common members of distinct reaction pathways. For this purpose, UniProtKB entry numbers of all proteins, which stood out in our proteome analysis [17], were entered in a Search Tool for the Retrieval of Interacting Genes/Proteins (STRING). STRING is an open-access tool available online (www.string-db.org) for the analysis of genes or proteins regarding their interactions. The program uses four different sources to quantitatively integrate interactions from a list of proteins or genes [21]. Applying the program, we found protein interaction groups containing proteins that we had detected in thyroid cells [17]. Interesting groups of interacting proteins presented by the computer program were selected and re-evaluated. The networks of interaction delivered by the STRING program demonstrate how punctual alterations caused by microgravity may propagate downstream in pathways.

## 2. Results and Discussion

### 2.1. Time-Dependence of Protein Expression

**Figure 1:**
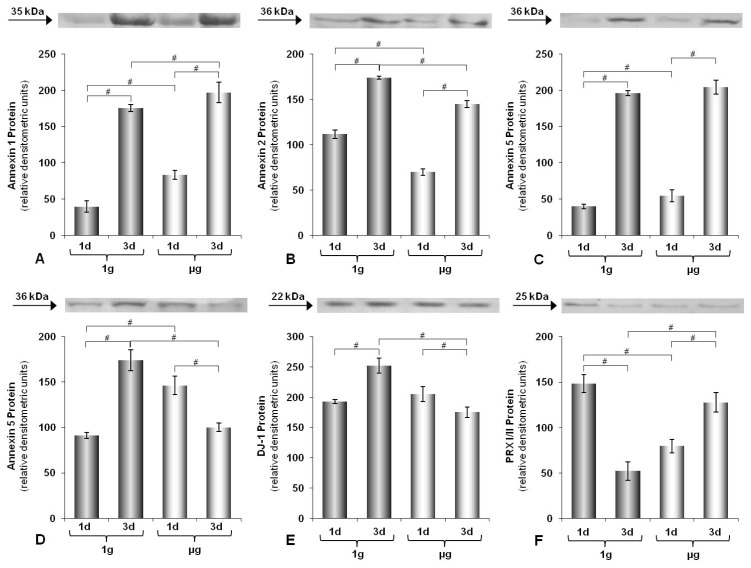
Western Blot analyses of various proteins of CGTH W-1 (**A**–**C**) and FTC-133 cells (**D**–**F**).

In order to investigate the effects of annulling gravity, we grew thyroid cell monolayers until sub-confluence. Then we randomized the culture flasks, filled them completely with culture medium without air bubbles and mounted one half of the flasks on the RPM, while the other half (static controls) was cultured in a normal laboratory incubator. After one or three days of continued incubation, the cells were harvested and distinct proteins were quantified by Western blotting. The experiments indicated that specific proteins may be up- or down-regulated by simulated microgravity at day one and three (Figure 1A,B, respectively), while other proteins appear resistant against alteration of gravity conditions (Figure 1C, 3 days). In addition, simulated microgravity can also up-regulate a protein one day and later down-regulate it again or vice versa (Figure 1D–F). From these results, we concluded that simulated microgravity may affect the cells in a time-dependent manner and that predefining the time points of investigation is essential when the influence of simulated microgravity on cellular functions is studied.

In order to proceed with the investigation of microgravity-dependent regulation of protein expression, we tried to identify as many thyroid cell proteins, which could be potential candidates of regulation on day 3, as possible. Therefore, we re-evaluated a proteome study performed on the human thyroid cell lines HTU-5, CGTH W-1, and FTC-133 [17]. Earlier, the proteome data had been evaluated looking only at proteins whose SDS-PAGE migration behavior corresponded to their known molecular weights [17]. Now we extended the analysis and included also proteins determined with Mascot scores above 100, but detected in gel pieces suggesting a smaller molecular weight than normally assigned to this protein. This step of extending the analysis appeared justified after we had seen that proteolytic cleavage during sample preparation changed the SDS migration behavior of proteins, but antibody binding and the examination of peptides had revealed a protein’s identity [18,22]. Re-evaluating the proteome analysis mentioned above [17] according to the new criteria, we recognized 533 proteins in FTC-133 cells, 337 in CGTH W-1 cells and 256 in HTU-5 cells. Only 82 proteins were found in all three cell lines, as the sites, where pieces were excised from SDS-gels, were not strictly identical when FFE separated proteins of the different cell lines were prepared for mass spectrometry [17]. All identified proteins were characterized by Mascot scores and emPAI values and labeled with their unique Universal Protein resource knowledgebase entry number (UniProtKB entry numbers, www.uniprot.org) as well as with the gene name according to the UniProtKB entry. Mascot scores and emPAI values were used to quantify individual proteins [17–20].

### 2.2. STRING Network Analysis

The UniProtKB entry numbers of the proteins were used to study the interaction behavior of the proteins identified. For this purpose, we entered the UniProtKB entry numbers of the detected proteins from each cell line into the STRING 9.0 network generation program (www.string-db.org) [21]. Executing the program loaded with the UniProtKB entry numbers of the 533, 337, and 256 proteins which we previously detected in the FTC-133, CGTH W-1, and HTU-5 cells, respectively, revealed that several of the detected proteins had interaction partners in each cell line.

**Figure 2:**
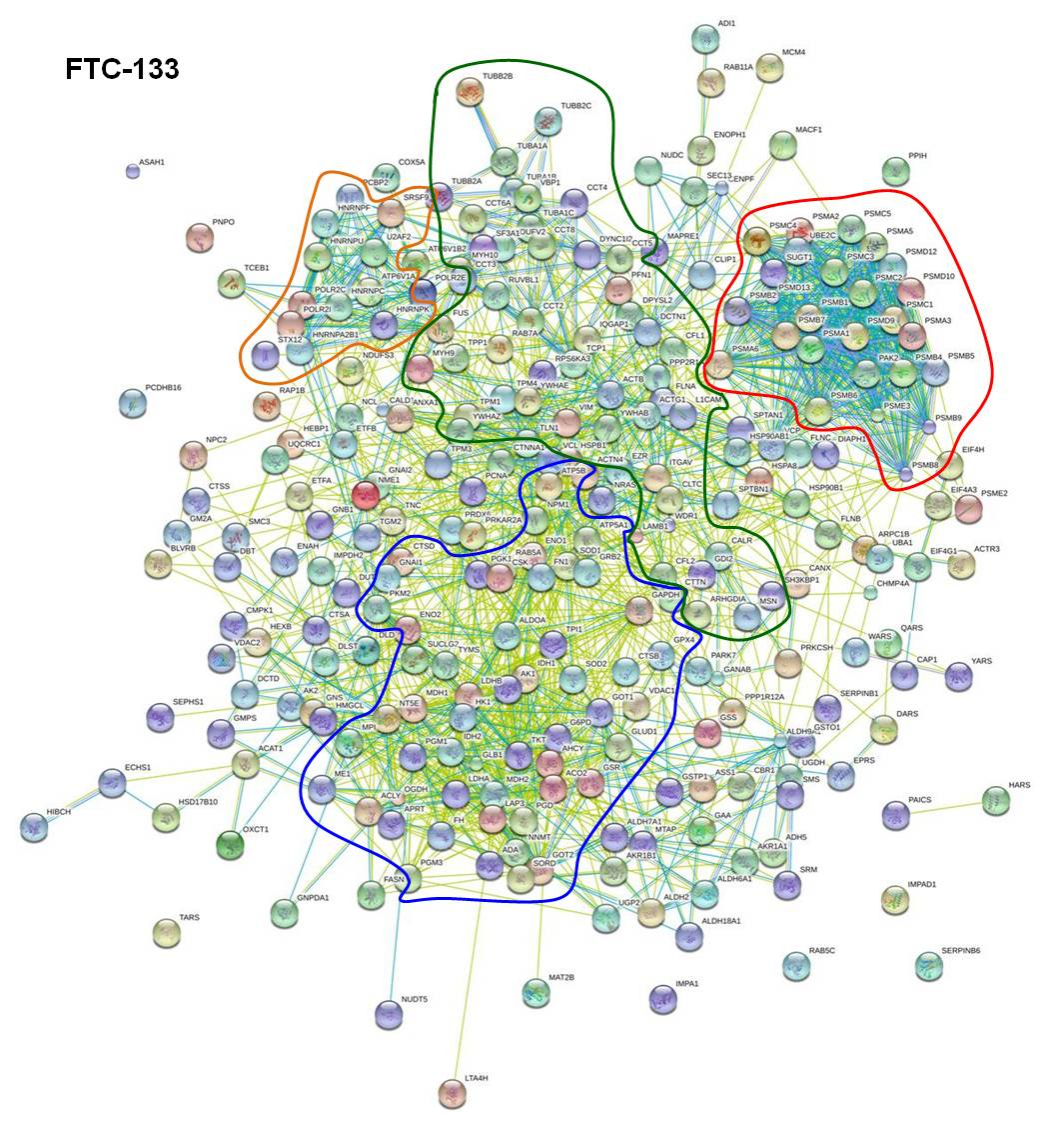
STRING (Search Tool for the Retrieval of Interacting Genes/Proteins) network analysis of the 274 interacting proteins found for FTC-133 cells. The interactions are shown in confidence view. Thicker lines represent a stronger association. The proteins are identified by their gene names located near each sphere.

**Figure 3:**
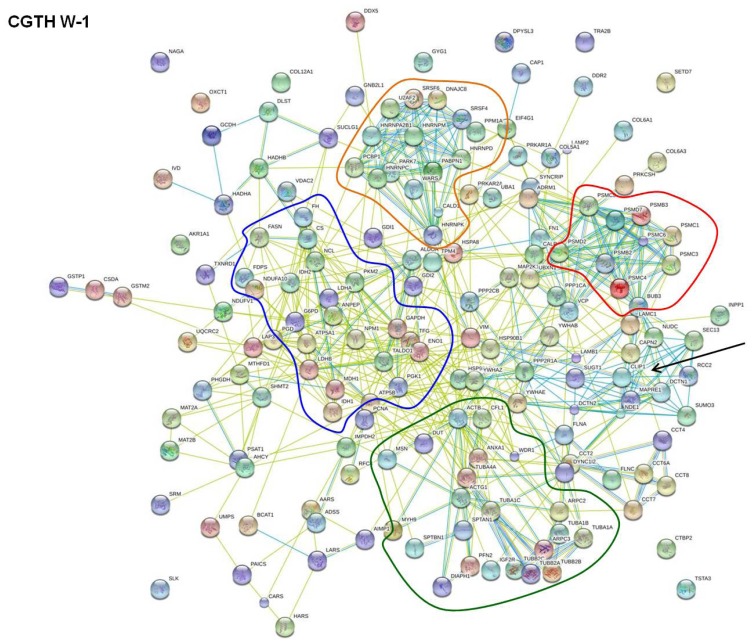
STRING network analysis of 172 interacting proteins found for CGTH W-1 cells. The interactions are shown in confidence view. Thicker lines represent a stronger association. The proteins are identified by their gene names located near each sphere.

**Figure 4:**
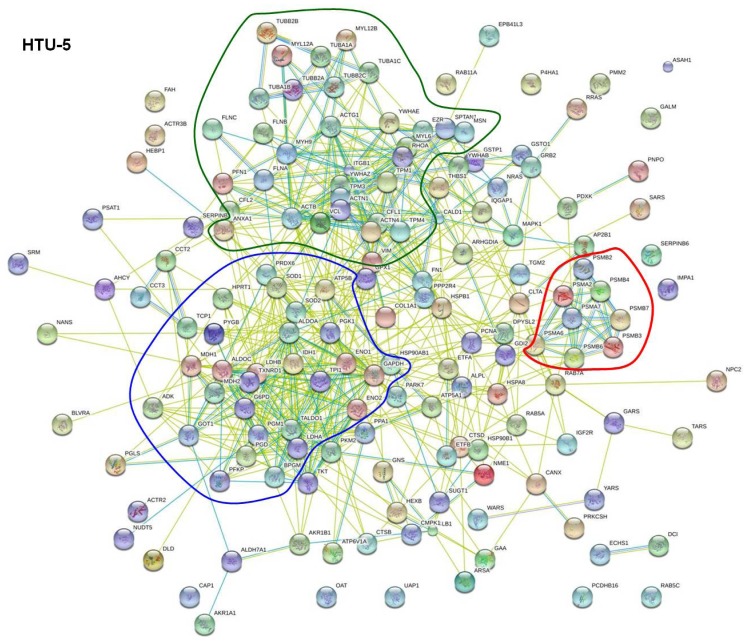
STRING network analysis of the 160 interaction proteins found for HTU-5 cells. The interactions are shown in confidence view. Thicker lines represent a stronger association. The proteins are identified by their gene names located near each sphere.

In order to discern strongly interacting proteins, we used the database view of STRING. This database view listed interaction groups for each cell line to which a part of the 533, 337, and 256 proteins mentioned above belong. For each protein interaction group, all proteins belonging to this group could be displayed. Amongst these, the proteins entered into the program were highlighted. When 10 or more proteins that we detected by our proteome analyses were found within a protein interaction group and these proteins comprised at least 10% of all proteins of the respective group, the identified thyroid proteins were considered as interacting proteins. This way we determined 274, 172, and 160 interacting proteins for FTC-133, CGTH W-1, and HTU-5 cells, respectively. They were summarized and re-analyzed (Figures 2, 3 and 4). Table 1 gives an overview on the number of interacting proteins found in the various cell lines and retrieved by using the database view of STRING. In addition, it displays functionalities which most of the interacting proteins exhibit. Split in functionalities, the numbers of interacting proteins appear higher for each cell line than indicated above, because many interacting proteins counted under “Regulation” show an additional functionality.

Figure 2 shows the interaction behavior of 274 interacting proteins of FTC-133 cells in detail. The STRING program revealed a complicated network of interaction of the 274 analyzed proteins, which comprise 51% of all in FTC-133 cells detected proteins (Figure 2). In addition, it indicates clusters of interacting proteins, which are circled by colored lines. There are clusters of interacting proteins involved in carbohydrate metabolism (Figure 2, confined within the blue circle), in protein formation (orange circle), in protein degradation (red circle), and in maintaining cell structures (dark green circle). The fifth group of interacting proteins (Table 1, Regulation), seen in the database mode of the STRING program, is not represented by a cluster in this picture.

These proteins are regulating various cell functions. They are distributed over the whole network and appear in the neighborhood of the regulated proteins. For example, mitochondrial ATP synthase, which consists of several subunits with gene names such as ATP5B and ATP5A1, is a member of the mitochondrial respiratory chain. It is involved in synthesis of ATP and simultaneously is regulating respiration by interacting with lactate dehydrogenase. In addition, it has relationships to both, the cytoskeletal protein moesin and the heat shock protein HSP90 (best seen in Figure 3). Moesin (MSN) and ezrin (EZR, shown in Figures 3 and 4) may be considered as regulatory and cytoskeletal proteins. They mediate actin-membrane linkages [23,24], regulate the organization and dynamics of the actin cytoskeleton in general [25], and influence the Rho/Rac GTPase signaling cascade [26]. Un-phosphorylated ezrin is able to initiate cell-cell contacts. These contacts are disrupted once ezrin is phosphorylated. Another regulating protein is the protein DJ-1 (PARK7) that facilitates cell survival under oxidative and mitochondrial stress [27]. Low protein DJ-1 content has been reported to lead to the disruption of the actin cytoskeleton [28] and may thus support the reshaping of cells during spheroid formation in microgravity. This protein is located between the clusters of carbohydrate metabolism and cell structuring and obviously influences proteins of both clusters. Furthermore, the 14-3-3 proteins scatter over all clusters. They comprise a group of seven isoforms in mammals which interact with numerous cellular proteins as well as with proteins involved in cellular functions, such as cell shaping by structural and cytoskeletal proteins, fatty acid synthesis, transcription, and protein folding. The most common function associated with these proteins is the sequestration and thus the inhibition of the function of these proteins [29].

Figure 3 shows the interaction characteristics of the 172 interacting proteins, which comprise 51% of all proteins found in CGTH W-1 cells. Four kinds of interacting proteins are indicated by their gene names. Two of them form rather strongly associated clusters. They include enzymes of protein formation (Figure 3, included in orange circles) and degradation (red circles). In addition, interacting proteins involved in carbohydrate metabolism (blue circles) or in maintaining cell structures (dark green circle) are indicated. Proteins of regulation are distributed within the other four clusters. However, a focus of accumulation became apparent around the proteins indicated by the gene names SEC13, MAPRE1, RCC2, CAPN2, NUDC (black arrow). Interestingly, these proteins regulate growth and proliferation, especially of tumor cells by modulating microtubules [30–34].

Figure 4 shows 160 (*i.e.*, 63%) of the proteins found in the normal thyroid cell line HTU-5, which were considered to be interacting proteins. These interacting proteins form clear clusters of enzymes involved in carbohydrate metabolism (circled by a blue line) and in cell structure maintenance (circled by a dark green line). In addition, a small number of enzymes involved in degradation were accumulated as indicated by the red circle, while a few enzymes involved in protein formation, such as some t-RNA synthetases were scattered. The group of proteins exhibiting regulatory activities is abundant as indicated in Table 1. They are distributed over the entire network and a focus of accumulation is not apparent.

### 2.3. Possible Ways of Signal Propagation

Figures 2, 3 and 4 showed that many proteins of each cell line can establish interaction with one or more other proteins. Thus, it appears reasonable that there are numerous proteins forwarding signals, which they receive when gravitational forces are removed. To the best of our knowledge tubulin is the only protein whose interaction behavior is affected directly when cells are exposed to simulated microgravity [35]. In our proteome study, we detected 9 different kinds of tubulins. Six of them were found in all three of the studied thyroid cells. They showed a strong interaction amongst themselves (Figures 2, 3 and 4, dark green circles), but they also interact with other cytoskeletal proteins. Important interaction partners are a group of proteins often observed as altered under microgravity, such as cell adhesion molecules and molecules involved in maintaining and remodeling the actin cytoskeleton [36,37]. They comprise of actin molecules (ACTB, ACTG1), filamin A (FLNA), non-muscular myosins (MYH9), and integrin-beta (ITGB1), which interact with fibronectin (FN1), cofilin (COL1), ezrin (EZR), and moesin (MSN) (Figures 2, 3 and 4). Thus, it appears reasonable that a microgravity-induced alteration of tubulins may for example be signaled to enzymes of the carbohydrate metabolism, since links are described in the literature between tubulin and actin, actin and cofilin as well as between cofilin and triose phosphate isomerase [38–40].

Taken together, the study shows that the STRING program may be applied to extract more information from proteome data [41–43]. Although some concerns have been published about this program [44], it proved useful for a deep evaluation of proteins, whose expression in thyroid cells has been detected when the cells were analyzed by FF-IEF and mass spectrometry. Similar clusters of proteins, strongly interacting within functionally related groups, were observed for all analyzed thyroid cell lines, even though the proteome analyses were carried out independently. Furthermore, chains of interaction were recognized, which show clearly how gravity-sensitive proteins, such as e.g., tubulin [35], can transfer the signal towards many other proteins.

## 3. Experimental Section

### 3.1. Culture of Different Thyroid Cell Lines

Three human thyroid cell lines were used. HTU-5 cells were derived from a normal human thyroid gland. These cells constitutively produce thyroglobulin and show normal diploid chromosome numbers. The cells were cultured in Coon’s F-12 medium supplemented with several growth factors as previously described [45]. The two other cell lines represented different types of follicular thyroid cancer. The FTC-133 cell line was isolated from a mediastinal lymph node metastasis of a follicular thyroid tumor [46], whereas the CGTH W-1 cell line originated from a metastasis in the sternum [47]. The two follicular thyroid carcinoma cell lines were cultured in RPMI-1640 medium containing 10% FCS, 100 U/mL penicillin, and 100 μg/mL streptomycin (all from Biochrom AG, Berlin, Germany).

Cells of each line were seeded into 36 T-25 cm^2^ culture flasks respectively and grown to subconfluent monolayers. Half of the culture flasks (*n* = 18) of each cell line were incubated further in a normal laboratory incubator at 37 °C and 5% CO_2_, while the other half (*n* = 18) was incubated on a desktop Random Positioning Machine (RPM, Dutch Space, an EADS company, Leiden, The Netherlands) after filling the T-25 culture flasks with medium completely. The RPM, positioned in an incubator adjusted to 37 °C and 5% CO_2_, was operated in a random walk (basic mode) which resulted in a residual sedimentation force of 10^−2^ g or lower within a maximal distance of 10 cm to the center of rotation, as previously described [5]. Cell samples were collected at day one and three from all cell lines.

After the various incubation periods, the cells were harvested, flash frozen and stored at −80 °C until further use. Three T-25 cm^2^ culture flasks were combined to one sample each, resulting in a replicate of *n* = 6 for each cell line and each incubation condition. For Western blot analysis, further cell samples were collected of each group (*n* = 5).

### 3.2. Proteome Analysis—FF-IEF and Mass Spectrometry

The procedure for proteome analysis was described previously [17,18,20]. Briefly, thawed cell samples were first suspended in HEPES buffer (10 mM HEPES, 15 mM MgCl_2_, 10 mM KCl, and 0.2% DTT) containing protease inhibitors and sonicated. Dissolved proteins were subjected to free-flow electrophoresis (FFE) using a BD™ FFE System (BD Diagnostics, Munich, Germany) with a stable pH gradient between pH 3 (anode) and pH 10 (cathode) [17,20]. After this first separation according to the isoelectric point, fractionated proteins were subjected to SDS-PAGE. Following electrophoresis, the gels were stained with Coomassie Brilliant Blue G-250 (BioRad, Munich, Germany), the bands of interest cut out of the gel and the proteins digested. The proteins of each gel band were analyzed using a nano-LC-MS/MS. The mass spectra obtained were used to identify the corresponding peptides/proteins by the MASCOT™ algorithm (version 2.1.6) [48]. A protein was considered identified when the cumulative score was at least 100.

### 3.3. STRING 9.0 Network Analysis

The identified proteins were then tabulated. For each protein, the UniProtKB entry number and gene name was acquired in UniProtKB and these names were used for the network generation with STRING 9.0 (www.string-db.org) [21]. The UniProtKB entry numbers were inserted in the input form “multiple proteins” and “*Homo sapiens*” was selected as organism. The resulting network view as well as the optional database view was downloaded. The database view resulted in a list of protein interaction groups, which were sorted by hand according to their size and their content of proteins detected by our proteome analysis. Proteins considered to be interacting proteins according to the criteria described in section 2.2 were summarized and entered in STRING 9.0 again.

### 3.4. Western Blot Analysis

For each Western blot, whole cell lysates were used. The gel electrophoresis, the trans-blotting, and densitometry were carried out following routine protocols as described earlier [49–51]. An equal amount of 10 μL of lysate containing 4 μg/μL proteins was loaded onto SDS-PAGE gels for each sample. Each Western blot was performed five times. Anti-annexin-1, anti-annexin-2, anti-annexin-5, and anti-DJ-1 protein were applied at a dilution of 1:1000 (Cell Signaling Technology, Inc., Danvers, MA, USA); anti-perioxiredoxin I/II antibodies were used at a dilution of 1:200 (Santa Cruz Biotechnology Inc., Santa Cruz, CA, USA). The secondary antibody (AP-linked) was utilized at a dilution of 1:3000 (Cell Signal Technology, Inc., Danvers, MA, USA). The use of a loading control was not feasible as there is no protein known so far which is not altered by annulled gravity. Therefore, equal protein amounts (40 μg in 10 μL) were loaded in each well as described above.

### 3.5. Statistical Analysis

Statistical analysis was performed using SPSS 16.0 (SPSS, Inc., Chicago, IL, USA, 2007). All data are expressed as means ± standard deviation (SD). Differences were considered significant at the level of *p <* 0.05 and marked by a #.

## 4. Conclusions

Changes in protein expression induced by cell cultivation on a RPM (*i.e.*, under simulated microgravity) happen during the first three days. However, so far it is unknown whether a protein is directly affected by annulling gravity or whether is influenced secondarily by a signal coming from an upstream protein reacting to the gravity directly. In this study, we used the STRING program to evaluate the proteome analysis performed on the thyrocyte cell line HTU-5 and the poorly differentiated follicular thyroid cancer cell lines FTC-133 and CGTH W-1 [17]. The program revealed that a number of proteins detected in each thyroid cell line are incorporated in a network of interacting proteins. The network includes clusters of strongly interacting enzymes involved in carbohydrate metabolism, protein formation, degradation, and cell shaping (Figures 2, 3 and 4) and proteins regulating cell growth. In addition, there are proteins that interact with members of two or more clusters. This way, chains of interacting proteins can be recognized linking proteins with different functionalities. Thus, it appears reasonable that a directly gravity-sensitive protein, such as e.g., tubulin [35], may initiate the alteration of a whole battery of enzymes via interacting proteins.

## Figures and Tables

**Table 1:** List of five topics of functionalities to which the various kinds of interacting proteins were allocated according to their functional properties.

Functionality of the interacting proteins	Found in FTC-133 cells	Found in CGTH W-1 cells	Found in HUT-5 cells
Carbohydrate metabolism	106	53	63
Maintaining cell structure	73	43	58
Protein synthesis/transcription	38	32	14
Degradation	28	13	8
Regulation	109	70	64
